# The Role of PPAR Ligands in Controlling Growth-Related Gene Expression and their Interaction with Lipoperoxidation Products

**DOI:** 10.1155/2008/524671

**Published:** 2008-07-06

**Authors:** Giuseppina Barrera, Cristina Toaldo, Stefania Pizzimenti, Angelo Cerbone, Piergiorgio Pettazzoni, Mario Umberto Dianzani, Carlo Ferretti

**Affiliations:** ^1^Dipartimento di Medicina e Oncologia Sperimentale, Sezione di Patologia Generale, Corso Raffaello 30, 10125 Torino, Italy; ^2^Istituto di Ricerche Biomediche “A. Marxer” RBM Merck Serono, Via Ribes 1, 10010 Colleretto Giacosa (Torino), Italy; ^3^Dipartimento di Anatomia, Farmacologia e Medicina Legale, sezione di Farmacologia, Via P. Giuria 13, 10125 Torino, Italy

## Abstract

Peroxisome proliferators-activated receptors (PPARs) are ligand-activated transcription factors that belong to the nuclear hormone receptor superfamily. The three PPAR isoforms (*α*, *γ* and *β*/*δ*) have been found to play a pleiotropic role in cell fat metabolism. Furthermore, in recent years, evidence has been found regarding the antiproliferative, proapoptotic, and differentiation-promoting activities displayed by PPAR ligands, particularly by PPAR*γ* ligands. PPAR ligands affect the expression of different growth-related genes through both PPAR-dependent and PPAR-independent mechanisms. Moreover, an interaction between PPAR ligands and other molecules which strengthen the effects of PPAR ligands has been described. Here we review the action of PPAR on the control of gene expression with particular regard to the effect of PPAR ligands on the expression of genes involved in the regulation of cell-cycle, differentiation, and apoptosis. Moreover, the interaction between PPAR ligands and 4-hydroxynonenal (HNE), the major product of the lipid peroxidation, has been reviewed.

## 1. THE ROLE OF PPAR IN CONTROLLING GENE TRANSCRIPTION

Peroxisome proliferator-activated receptors (PPARs) are members of the steroid hormone nuclear receptor superfamily which act by altering the transcription of PPAR-regulated genes by means of a recognition sequence known as a peroxisome proliferation responsive element (PPRE) [1].

The term peroxisome proliferator-activated receptor is derived from early observations in rodent livers that certain industrial compounds could cause an increase in size and number of peroxisomes [2, 3]. Subsequently, these compounds, including fibrates, were found to bind to certain recently identified nuclear receptors [4]; hence, the term “PPAR” arose. PPAR agonists are not known to induce peroxisome proliferation in primates or humans, making the term PPARs archaic as well [5]. At least three subtypes of PPARs have been identified: PPAR*α*, the first isolated from mice liver in 1990 by Issemann and Green [4] and involved in fatty acid oxidation; PPAR*γ*, identified by Tontonoz and collaborators as a transcription factor associated with adipocyte determination and differentiation [6]; and PPAR*β*/*δ*, ubiquitously expressed and involved in basic cellular functions [7, 8]. Like other steroid hormone nuclear receptors, PPARs contain several modulating domains: a ligand binding domain (LBD) to which the specific PPAR agonist binds; a transactivating domain (activation function 2, AF 2), which undergoes conformational changes, in response to ligand binding, allowing the heterodimerization with RXR and facilitating recruitment of coactivators and release of corepressor; and finally a DNA-binding domain, which interacts with PPRE [3, 9–11].

**Figure 1:**
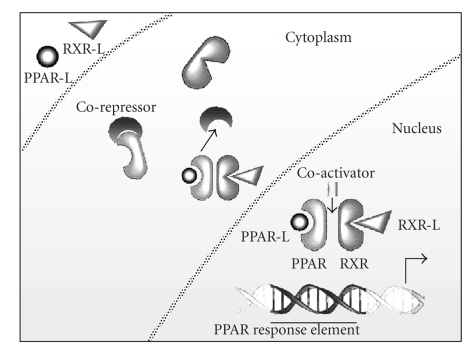
Mechanism of PPAR action. PPARs in response to ligand binding in the cytosol, dimerize with RXR, recruit coactivators and release corepressor; in the nucleus a multimolecule complex, formed by PPAR, PPAR ligand, RXR, RXR-ligand, and accessory proteins bind PPRE DNA sequences in the promoters of target genes.

PPAR coactivator and corepressor are small accessory molecules that are critical determinants of the transcriptional complex. These accessory molecules include coactivator proteins, like PPAR*γ* coactivator-1 (PGC-1); steroid receptor coactivator and CREB (cAMP-response element binding protein)-binding protein, recruited from the activated PPAR; and corepressor proteins, like nuclear receptor corepressor (N-CoR) and silencing mediator for retinoid and thyroid hormone receptors (SMRTs1), released upon PPAR activation [12, 13]. A multimolecule complex formed by PPAR, PPAR ligand, RXR, RXR-ligand (purportedly 9-cis-retinoic acid) and accessory proteins ultimately combine to cause the PPAR response through the binding with PPRE sequences consisting of a direct repeat of the consensus half-site motif (AGGNCA) spaced by a single nucleotide [14] (Figure 1).

Several genes that are selectively upregulated by a given PPAR isotype have been identified over the years and a majority of these genes is known to play a central role in energy metabolism. Moreover, microarray technology and genome wide identification of PPREs suggest the existence of many other target genes that were not previously known to be regulated by PPAR. The identified PPRE putative sequences on target genes for PPARs are listed in Table 1.

Recent evidence indicates that the PPAR response can result both in gene activation and repression. As far as it regards gene repression, PPAR*γ* was shown to be unable to bind to DNA while it is associated with the corepressor complex. In contrast to PPAR*γ*, the interaction between NCoR/SMRT and PPAR*β*/*δ* does not impair its DNA binding [54, 55]. PPAR*γ*, after ligand binding, dissociates from the corepressor, and binds to DNA via PPREs. The liberated corepressor protein SMRT interacts with the signal transducer and activator of transcription-3 (STAT3), which inhibits STAT-dependent transactivation [56]. Recent data suggest that PPAR*γ*-mediated transrepression may involve stabilization of corepressor recruitment after posttranslational PPAR modification by sumoylation [57].

In macrophages, PPAR*β*/*δ* was shown to function as an activator of the monocyte chemoattractant protein (MCP-1) gene by sequestering a transcriptional repressor, specifically the transcriptional repressor B-cell lymphoma-6 (BCL-6) [37, 58]. The ligand-induced activation of PPAR*β*/*δ* releases the corepressor BCL-6, which is thought to inhibit MCP-1 expression. Hence, PPAR*β*/*δ* can function as an intrinsic transcriptional repressor, a mechanism that is also shared by other nuclear receptors such as the thyroid hormone receptor (NR1A1, NR1A2), retinoic acid receptor (NR1B1, NR1B2, NR1B3), Rev-Erb (NR1D1, NR1D2) and COUP-TF (NRT2F3).

The best-documented mechanism by which PPAR*α* can transrepress non-PPREs containing genes is its ability to physically interact with the p65 subunit of nuclear factor (NF)-*κ*B, which inhibits NF-*κ*B-dependent transactivation [59]. However, PPAR*α* activators do not inhibit all NF-*κ*B-driven target genes and their effect is promoter context-dependent. Taken together, data obtained about PPAR transcriptional regulation demonstrated that PPARs can also modulate the transcriptional activity of non-PPRE containing genes via transrepression.

## 2. PPAR LIGANDS

PPAR ligands are a heterogeneous group that includes both endogenous and exogenous ligands [60]. Activating ligands for PPARs are semiselective for the subtype and selectivity depends on ligand concentration and cell type. Endogenous ligands include unsaturated fatty acids that bind all three PPARs, with PPAR*α* exhibiting the highest activity, while saturated fatty acids are weak PPAR ligands in general [61]. Biological modifications of linoleic acid, linolenic acid, eicosapentanoic acid (EPA), and arachidonic acid originate PPAR*α* activators [62–64]. Moreover, the oxidized form of EPA, eicosanoids (15-hydroxy-eicosatetranoic acid, HETE and HODEs), and leukotriene B4 has also been reported to be PPAR*α* activators [62–66].

The natural ligands of PPAR*γ* include several prostanoids such as 15-deoxy-prostaglandin J2 (15d-PG J2) and 15-hydroxy-eicosatetranoic acid (HETE), which are metabolites of arachidonic acid [67]. 15d-PG J2 (the most widely used natural ligand for PPAR*γ*) is gamma-selective at low concentrations but also activates alpha at higher levels [68, 69]. Like PPAR*α*, PPAR*β*/*δ* is activated by long chain fatty acids, including several polyunsaturated fatty acids and eicosanoids [3]. Erucic acid has been reported to be more selective for PPAR*β*/*δ* than other PPAR subtypes [70].

Synthetic ligands of PPARs have been demonstrated to possess pharmacological activity. The triglyceride-lowering/high-density lipoprotein (HDL)-raising fibrates (gemfibrozil, fenofibrate, clofibrate, ciprofibrate) are PPAR*α* agonists used clinically to treat dyslipidemia [71, 72]. The insulin-sensitizing thiazolindinedione (TZD) class (troglitazone, pioglitazone and rosiglitazone) is PPAR*γ* activators that are used to treat diabetes mellitus [73, 74]. Several nonsteroidal anti-inflammatory drugs (NSAIDs), in particular indomethacin and ibuprofen, bind to PPAR*γ* and are weak PPAR*γ* agonists at high micromolar concentrations [75, 76].

The first PPAR*β*/*δ*-selective agonists (L-165041 and GW501516) were shown to augment HDL-C in diabetic mice as well as in obese rhesus monkeys, in which they decreased elevated levels of triglycerides and insulin [77, 78].

## 3. THE ROLE OF PPAR LIGANDS IN AFFECTING CELL PROLIFERATION AND DIFFERENTIATION

Although a direct control of PPAR transcription is limited to a very small number of growth-related genes (see Table 1), the ability of PPAR ligands to inhibit cell growth by inducing cell differentiation or apoptosis has long been demonstrated in several cell lines. In general, the PPAR*α* and the PPAR*γ* ligands display an inhibitory effect on cell growth, while PPAR*β*/*δ* have different effects, strictly dependent on the cell type. Indeed, in murine colorectal cells, the Apc-*β*-catenin tumour-suppressor pathway was shown to repress PPAR*β*/*δ* expression [79]. More recently it was suggested that ligand activation of PPAR*β*/*δ* induces expression of cyclooxygenase-2 (COX2), which could theoretically promote cell growth and inhibit apoptosis through mechanisms that involve the production of prostaglandins and/or inflammation-dependent signalling [80]. However, there are several observations that are inconsistent with the idea that ligands of PPAR*β*/*δ* potentiate cell growth. For example, inhibition of cell growth is observed in a variety of different cells and cell lines cultured in the presence of highly specific PPAR*β*/*δ* ligands including human colonocytes [81], a human lung adenocarcinoma cell line [82], mouse lung fibroblasts [83], rat cardiomyocytes [84], a human keratinocyte cell line [85], normal human keratinocytes [86], and mouse primary keratinocytes [87]. Some evidence about the effects of PPAR ligands on cell differentiation, cell cycle progression, and apoptosis induction is illustrated as follows.

### 3.1. Effect of PPAR ligands in differentiation induction

The first demonstration of PPAR*γ* involvement in adipocyte differentiation was given by Tontonoz et al. (1993) [6]. Subsequently, PPAR*γ* and PPAR*α* ligands have been demonstrated to induce differentiation alone or in association with other differentiation inducers. It has been demonstrated that clofibrate, a PPAR*α* ligand, increases the differentiation of HL-60 cells induced by retinoic acid and all-trans retinol [88]. Other PPAR*α* activators, including putative endogenous ligands such as fatty acids, induce differentiation and inhibit proliferation in keratinocytes [89]. The PPAR*α* ligand, ciprofibrate induces differentiation of HL-60 cells and its effect is potentiated by phorbol 12-myristate 13-acetate (TPA) [90]. Benzafibrate induces differentiation of HL-60, U937, and K562 cells [91]. PPAR*γ* ligands induce terminal differentiation of human liposarcoma cells “in vitro” and in patients suffering from advanced liposarcoma [92], and promote terminal differentiation of malignant breast epithelial cells “in vitro” [93]. Our research group demonstrated that both PPAR*α* (clofibrate and ciprofibrate) and PPAR*γ* ligands (troglitazone and 15d-PG J2) inhibit growth of HL-60 human leukemic cells and induced the onset of monocytic like differentiation [94]. In another leukemic cell line, U937 cells, PPAR*γ* ligands inhibited proliferation but did not induce differentiation (except the higher doses of 15d-PG J2 which induced a poor monocytic differentiation) [94] indicating that the differentiation induction by PPAR ligands is cell-type specific.

Several experimental results indicate that ligand activation of PPAR*β*/*δ* induces terminal differentiation of keratinocytes [86, 87, 95, 96] and it has also been shown that differentiation of breast and colon cancer cell lines is associated with increased expression of PPAR*β*/*δ* [97]. PPAR*β*/*δ* expression also increases following differentiation in human primary macrophages or in monocyte/macrophage cell lines [98]. In addition, activation of PPAR*β*/*δ* using a selective agonist promotes oligodendrocyte differentiation in a mouse cell culture [99].

### 3.2. Effect of PPAR ligands on cell cycle progression

Evidence has been demonstrated that PPAR ligands inhibit cell growth by acting on cell cycle progression. Fibrates, in a dose dependent-manner, significantly alter the cell cycle distribution, mainly leading to G0/G1 phase increase and a G2/M phase reduction in human leukemic cell lines [91]. In HL-60 human leukemic cells, both PPAR*α* and PPAR*γ* ligands increase the proportion of G0/G1 cells [100]. PPAR*γ*, ectopically expressed in nonprecursor fibroblastic cell lines, induces the conversion to adipocytes and induces the expression of p21 and p18, two cyclin/cyclin-dependent kinase (CDK) inhibitors [101]. Troglitazone arrests U937 cells in the G1 phase of the cell cycle [102] and inhibits cyclin D1 expression in MCF7 cells [103]. PPAR*γ* activation induces cell cycle withdrawal of preadipocytes via suppression of the transcriptional activity of E2F/DP DNA-binding complex [104]. E2F activity is regulated by the tumour suppressor retinoblastoma protein (pRb) that, when hypophosphorylated, binds and inactivates the E2F transcription factor [105]. Interestingly, PPAR*γ* ligands inhibit pRb phosphorylation in vascular smooth muscle cells [106–108], increasing the amount of hypophosphorylated pRb able to bind E2F. Others found that troglitazone inhibits the growth of six of nine pancreatic cancer cell lines, by inducing G1 phase cell cycle arrest through the up-regulation of the expression of p21 [109].

Ligand activation of PPAR*β*/*δ* with GW0742 prevents cell cycle progression from G1 to S phase and attenuates cell proliferation in N/TERT-1 keratinocyte cells [110].

### 3.3. Effect of PPAR ligands on apoptosis induction

Inhibition of cell proliferation by PPAR ligands is also supported by their effect on apoptosis induction. PPAR*γ* ligands seem to be more effective than PPAR*α* in inducing apoptosis, since its proapototic activity has been demonstrated in a wide variety of experimental cancer models [111]. PPAR*γ* ligands have been reported to reduce levels of FLICE-inhibitory protein (FLIP), and apoptosis-suppressing protein that blocks early events in TRAIL/TNF (Tumor necrosis factor-related apoptosis inducing ligand/Tumor necrosis factor) family death receptor signalling [112]. 15d-PG J2 and troglitazone suppress DNA synthesis and induce apoptosis in a dose-dependent way in HT-29 colon cancer cells, whereas ligands for PPAR*α* and *β*/*δ* had no significant effect [113]. Troglitazone inhibited growth of liver cancer cells PLC/PRF/5, HepG2 and HuH-7, by inducing apoptosis through caspase-3 activation [114]. In breast cancer cells, both troglitazone and 15d-PG J2 induce apoptosis [115, 116]. Kondo et al. have shown that the 15d-PG J2-induced accumulation of p53 results in the activation of a death-inducing caspase cascade mediated by Fas and the Fas ligand in neurons [117]. Activation of PPAR*γ* by troglitazone or 15d-PG J2 inhibits cell growth via apoptosis and blocks cell cycle in human colorectal cancer [118]. However, in some cell models, both PPAR*α* and PPAR*γ* displayed proapoptotic activity, as it has been demonstrated in the HL-60 cell line [100] and in the lymphoblastic leukaemia cell line [119]. In keratinocytes [120], ovarian cancer cells [121] and in human hepatoma cell line SK-HEP-1 [122], PPAR*α* ligands have been reported to induce apoptosis.

Colon cancer cell lines cultured in the presence of the PPAR*β*/*δ* ligand GW501516 exhibit inhibited levels of apoptosis [123, 124]. It has been postulated that apoptosis is inhibited by PPAR*β*/*δ*-dependent downregulation of the tumour suppressor phosphatase and tensin homologue deleted on chromosome ten (PTEN) and upregulation of the 3-phosphoinositide-dependent kinase-1 (PDK1) and integrin-linked kinase-1 (ILK1) [22]. The net effect of this change in activity would have increased phosphorylation of protein kinase B (Akt) and inhibition of apoptosis; and these changes were shown in cultured primary keratinocytes [22]. In mouse keratinocytes, PPAR*β*/*δ* inhibits proliferation and promotes cell survival and migration [96, 125, 126]. In contrast with these data, prostacyclin (PGI_2_) was shown to promote apoptosis in a kidney cell line, most probably through PPAR*β*/*δ* activation [127].

## 4. THE ROLE OF PPAR LIGANDS IN THE CONTROL OF GROWTH-RELATED GENE EXPRESSION

The effect of PPAR ligands in the expression of growth regulatory genes has been in part illustrated in the previous section. Results obtained until now do not allow the identification of a precise signalling pathway and the PPAR target genes that mediate the antiproliferative effects remain elusive, as genomic responses to PPAR*γ* activation in cancer cells are highly complicated [128]. PPAR*γ* ligands seem to be more effective than PPAR*α* ligands in inhibiting cell growth, thus the majority of data about the gene expression following treatment with PPAR ligands is obtained in PPAR*γ* ligand-treated cells. Recently, some evidence has been found for PPAR*β*/*δ* and its ligands in regulating gene expression. However, the number of growth-regulatory genes, affected by specific PPAR*β*/*δ* ligands, is limited and comprises growth-inducing genes such as COX2 [80], and Akt, via transcriptional upregulation of integrin linked kinase (ILK) and 3-phosphoinositide-dependent kinase-1 (PDK1) [22] and the decrease in the level of ERK phosphorylation [110].

Reported causal mechanisms for PPAR*γ* growth inhibitory effects include attenuated expression of protein phosphatase 2A and subsequent inhibition of E2F/DP DNA binding [129], the inhibition of cyclins D1 and E, inflammatory cytokines and transcription factors expression [130] and increased expression of an array of gene products linked to growth regulation and cell maturation [128]. Moreover, our and other research groups have demonstrated that the reduction of cell growth by PPAR ligands is accompanied by the downregulation of the c-myc gene in myeloid leukaemia cells [131] and in colon cancer cells [132, 133]. In the HL-60 cell line, both PPAR*α* (ciprofibrate and clofibrate) and PPAR*γ* (troglitazone and 15d-PG J2) ligands inhibit c-myb and cyclin D2 expressions [100]. In prostate cancer cells PPAR*γ* ligands omega-6 fatty acids and ciglitazone down-regulated (1-2-fold) beta-catenin and c-myc expression and the selective PPAR*γ* antagonist GW9662 abolished the effect of those ligands, demonstrating a PPAR-dependent mechanism. 15-d PG J2 inhibits N-myc expression in neuroblastoma cells [134] while it does not decrease c-myc expression in vascular smooth muscle cells [135].

The major part of the genes of which expression is modulated by PPAR*γ* ligands does not contain PPRE putative sequences in their promoter regions. Besides downregulation of c-myc, c-myb, and cyclin D2 genes, previously reported, an array ofnon-PPAR*γ* targets has been implicated in the antitumor activities of troglitazone and/or ciglitazone in several cell systems. These targets include intracellular Ca^2+^ stores [136], phosphorylating activation of extracellular signal-regulated kinases [137, 138], c-JunN-terminal protein kinase, and p38 [139], upregulationof early growth response-1 [140], the CDK inhibitors p27 [141] and p21 [142], the tumor suppressor protein p53 and the p53-responsive stress protein Gadd45 [135], and altered expression of B-cell leukemia/lymphoma 2 (Bcl-2)family members [139]. However, some of these targets appear to be cell-type specific due to differences insignalling pathways regulating cell growth and survival in differentcell systems.

Recent findings demonstrate that part of the above mentioned growth-regulatory genes are affected by PPAR ligands, mostly by PPAR*γ* through a PPAR-independent mechanism. The most important evidence of PPAR-independent effects displayed by PPAR ligands is illustrated in Table 2.

## 5. THE PRODUCTS OF LIPID PEROXIDATION IN THE CONTROL OF GROWTH-RELATED GENE EXPRESSION

Reactive intermediates produced during oxidative stressful conditions cause the oxidation of polyunsaturated fatty acids such as arachidonic, linolenic, and linoleic acids of membrane lipid bilayers or low-density lipoprotein [156] leading eventually to the formation of several aldehydes. Among the products of oxidative breakdown of polyunsaturated fatty acid, 4-hydroxy-2,3-trans-alkenals have been proposed as ultimate messengers of lipid peroxidation-induced injury, because they can diffuse from the site where they are produced and can reach different intracellular and extracellular targets [157–159]. 4-hydroxynonenal (HNE), the aldehyde most represented in the 4-hydroxy-2,3-trans-alkenal class, has long been investigated, since, at concentrations near to those “physiologically” found in normal cells and plasma, it modulates cellular functions, gene expression and biochemical pathways, without cytotoxic effects [160]. For this reason, HNE has been proposed by several authors as an intracellular signalling mediator, rather than a toxic product of lipid peroxidation [159, 161]. Previous results demonstrated the antiproliferative and differentiative effects of HNE in leukemic cells [162, 163] and the antiproliferative and proapoptotic effects in a number of different cell models [164, 165]. Deeper investigations into HL-60 cells showed that the proliferation block occurred at the level of the G0/G1 stage of the cell cycle [163]. Further experiments showed that the HNE effects depend on the inhibition of the cyclin expression, and especially of cyclins D2, D1, and A [166]. The reduction of cyclin expression can result in a reduced activity of cyclin/CDK complexes which principally regulate the phosphorylation of the pRb. In highly proliferating tumour cells, pRb is constantly in the hyperphosphorylated status. When hyperphosphorylated, pRB cannot bind to E2F transcription factors that can promote the G1/S cell cycle phase passage. After HNE treatment, pRb remains hypophosphorylated, and E2F remains bound to pRb [167]. HNE not only reduces the phosphorylation of pRb, but also decreases the amount of “free” E2F bound to the P2 c-myc promoter. These effects can explain the blocking of c-myc expression demonstrated in HNE-treated cells.

The hypophosphorylation of pRb proteins may depend on the inhibition of cyclin expression, however, this effect may also be related to the increase of the expression of p21, an inhibitor of the cyclin/CDK complexes, induced by HNE treatment [167]. Another effect of HNE, also important for cell multiplication, is that displayed on telomerase activity and hTERT expression. The activity of telomerase and the expression of its catalytic subunit hTERT, were inhibited by HNE in three different human leukemic cell lines, HL-60, U937 and ML-1 [168]. The binding studies of E-box in the hTERT promoter demonstrated that in HNE-treated HL-60 cells there is a decrease in Myc binding complexes and an increase in Mad-1 binding complexes which could contribute to the switch from c-Myc/Max to Mad1/Max with repressor activity of the transcription.

HNE is able to induce p53 expression in ML-1 cells, according to previous results demonstrating the induction of p53 expression by HNE in the SK-N-BE human neuroblastoma cell line [165]. Moreover, in SK-N-BE cells apoptosis was substantially increased even with 1 *μ*M HNE. At the same time, the expression of the p53 family members, p63 and p73, was strongly increased as well as the expression of the cyclin/CDK inhibitor p21 and the proapoptotic bax gene. Since p21 and bax are the two main targets for the transcription factor p53, these results indicate that HNE, by acting on p53 gene expression, can regulate the p53 target genes.

## 6. INTERACTION BETWEEN PPAR LIGANDS AND LIPOPEROXIDATION PRODUCTS

The relationship between oxidative stress-related molecules and PPAR activation has not yet been elucidated. Based upon their capacity to elicit cellular responses to a variety of stimuli, PPARs may represent a class of molecules which allow the biochemical adaptation to a diverse range of internal and external signals. These include oxidised LDL [169] and inflammatory agents as well as 15d-PG J2 [170] and leukotriene B4 [65]. However, other molecules generated during inflammation may be involved. In the cultured mesangial cells, PPAR*γ* is activated by various oxidative stress-related molecules such as TPA, TNF alpha, and H_2_O_2_ [171]. The physiological ligand of PPAR*γ*, 15d-PG J2, is a potential inducer of intracellular oxidative stress that mediates the cytotoxic effects in human neuroblastoma cells [172]. On the other hand, the activation of PPAR*α* leads to increased oxidative stress in liver cells [173]. On the basis of this link between oxidative stress and PPAR activation and between oxidative stress and lipoperoxidation induction, our research group investigated, for the first time, the interaction between the major lipoperoxidation product, HNE, and PPAR activation in HL-60 and U937 human leukemic cells [94]. We demonstrated that HNE increases the monocytic differentiation induced by the PPAR*α* ligand ciprofibrate, and PPAR*γ* ligands, troglitazone and 15d-PG J2, in HL-60 cells. Whereas, neither PPAR*α* nor PPAR*γ* ligands induce U937 differentiation. Moreover, in this cell line, only PPAR*γ* ligands reduce cell growth. HNE also significantly inhibits cell growth when given alone, and strengthens the growth inhibitory effect of a low dose of PPAR*γ* ligands. HNE promotes at the same time a great increase in the expression of PPAR*γ* in both HL-60 and U937 cells, without any modification of the PPAR*α* expression. These results suggest a synergistic effect of HNE and PPAR*γ* ligands in blocking cell growth and in promoting the differentiation in HL-60 cells.

More recently, we analysed the effects of PPAR*γ* ligands (rosiglitazone and 15d-PG J2) and HNE, alone or in association, on proliferation, apoptosis, differentiation, and growth-related and apoptosis-related gene expressions in CaCo-2, colon cancer cells. Results obtained indicate that, in this cell line, PPAR*γ* ligands and HNE inhibited cell growth and induced differentiation or apoptosis by different signalling pathways. The common feature consisted of the inhibition of c-myc expression, whereas the apoptosis was induced by 15d-PG J2 and HNE and, to a minor extent, by rosiglitazone and the differentiation was induced by rosiglitazone and by 15d-PG J2, but not by HNE. Moreover, HNE induced p21 expression, while PPAR*γ* ligands did not. Bax expression was increased by HNE and 15d-PG J2, but not by rosiglitazone. HNE did not induce an increase of PPAR*γ* expression and did not display synergism or antagonism towards PPAR*γ* ligands.

These various results, obtained in different cell models, strongly demonstrate that the gene expression control exerted by PPAR ligands is dependent on the cell type examined.

An interaction between HNE and PPAR*γ* has also been demonstrated by Muzio et al. (2006) [174]. These authors found that arachidonic acid induces suppression of human lung tumor A549 cell growth, increases lipid peroxidation and decreases aldehyde dehydrogenase 3A1 ALDH3A1, which may determine an accumulation of endogenous HNE. These phenomena are associated with the increased expression of PPAR*γ*, suggesting a relationship between endogenous HNE levels and PPAR*γ* expression. Moreover, it has been postulated that HNE can represent an endogenous modulator of PPAR*β*/*δ* activity, since HNE is an endogenous ligand for PPAR*β*/*δ* and activates PPAR*β*/*δ* target genes [175]. This datum suggest that the binding between HNE and PPAR*β*/*δ* can modulate PPAR*β*/*δ* activity in all cell types, since PPAR*β*/*δ* is ubiquitously expressed.

**Figure 2:**
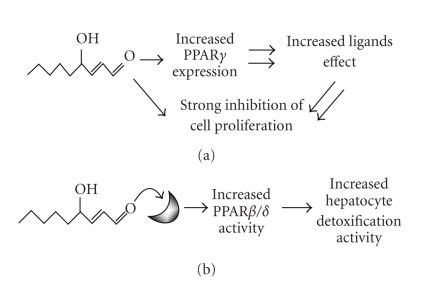
Different interactions between HNE and PPAR. (a) HNE increases PPAR*γ* expression in leukemic cell lines; (b) HNE binds and activates PPAR*β*/*δ*.

The different interactions between HNE and PPAR are summarized in Figure 2.

These findings represent an intriguing suggestion about the role played by the lipoperoxidation products in controlling cellular PPAR-dependent responses, not only regarding cell proliferation control but also in the regulation of different metabolic pathways, and indicate that the interaction between oxidative stress products and PPAR activity represents a new research field in expansion.

## Figures and Tables

**Table 1:** 

Genes containing PPRE putative sequences	Function of gene	Ref.
*Lipid metabolism*		
P450 4A6	Omega oxidation of fatty acids	[15]
malic enzyme gene	Fatty acid synthesis	[16]
apoA-I and apoA-II.	Components of HDL	[17]
LPL (lipoprotein lipase)	Hydrolysis of triglycerides	[18]
UCP3 (Uncoupling protein 3)	Fatty acid transport and thermogenesis	[19]
CEH (Cholesteryl ester hydrolase)	Hydrolysis of stored cholesterol esters in macrophage foam cells and release of free cholesterol for high-density lipoprotein-mediated efflux	[20]
Aox/ACO (Acyl-CoA oxidase)	Beta-oxidation in peroxisome	[21]
HD (enoyl-CoA hydratase/3-hydroxyacyl-CoA dehydrogenase)	Beta oxidation in perixisome	[21]
ILK (Integrin-linked kinase)	Integrin-mediated signaling	[22]
HMG-CoA (3-hydroxy-3-methylglutaryl coenzyme A synthase and reductase)	Cholesterol biosynthesis	[23]
LRP (lipoprotein receptor-related protein)	Lipoprotein metabolism, neurological function, tissue remodelling, protease complex clearance, cell signal transduction	[24]
CPT1beta (human carnitine palmitoyltransferase 1beta)	Fatty acid mitochondrial beta-oxidation	[25]
FABP (fatty acid binding protein)	Lipid transport (solubilization of long-chain fatty acids)	[26]
ADRP (Adipose differentiation-related protein)	Maintenance of lipid stores in non-adipocytes	[27]
FIAF (The fasting-induced adipose factor	Circulating lipoprotein lipase inhibitor secreted from adipose tissue	[28]

*Carbohydrate metabolism*		
betaGK (beta-cell-specific glucokinase)	Glucose-sensing apparatus in pancreatic beta-cells	[29]
GPDH (Glycerol 3-phosphate dehydrogenase)	NAD-dependent enzyme that catalyzes the oxidation of sn-glycerol 3-phosphate to dihydroxyacetone phosphate. It restores NAD+.	[30]
UGDH (UDP-glucose dehydrogenase)	Biosynthesis of complex carbohydrates and detoxification of toxic compounds in the liver	[31]
PDK (Pyruvate dehydrogenase kinase)	Modulation of pyruvate dehydrogenase complex activity	[32]
SHP (Small heterodimer partner)	Bile acid-dependent down regulation of gluconeogenic gene expression in liver	[33]

*Inflammation*		
Prm3 (thromboxane receptor (TP) beta promoter)	Thromboxane receptor (TP) beta transcription	[34]
IL-1ra (Interleukin-1 receptor antagonist)	IL-1 receptor signaling blockage	[35]
CD36 (scavenger receptor)	Scavenger receptor	[36]
sPLA2-IIA (Group IIA secretory phospholipase A2)	Proinflammatory effect	[37]
AhR (Aromatic hydrocarbon receptor)	Proinflammatory effect	[38]

*Growth factors and cell cycle regulators*		
SSAT (spermidine/spermine N1-acetyltransferase)	Polyamine catabolism	[39]
GOS2 (GO/G1 switch gene 2)	Cell cycle regulation	[40]
VEGF (Vascular endothelial growth factor)	Vasculogenesis	[41]
IGFBP-1 (Insulin-like growth factor-binding protein 1)	Binding protein of insulin-like growth factor (IGF)-I and IGF-II. Biomarker for metabolic and hyperproliferative diseases	[42]

*Detoxification and redox enzymes*		
CYP1A1 (Cytochrome P450 1A1)	Degradation of endobiotics and the bioactivation of numerous environmental procarcinogens	[43]
GST (glutathione S-transferase gene)	Antioxidant function	[44]
POX (Proline oxidase)	Redox enzyme	[45]
VDUP-1 (Vitamin D-upregulated protein-1)	Inhibition of thioredoxin-1 which plays a role in the regulation of cellular redox balance (Cellular redox balance)	[46]

*Others*		
BCM (Beta-carotene 15,15′-monooxygenase)	Vitamin A biosynthesis	[47]
I-BABP (Ileal bile acid-binding protein)	Enterohepatic circulation of bile acids	[48]
PCLN-1 (paracellin-1)	Tight-junction protein, exclusively, in the kidney	[49]
BACE1(Beta-site amyloid precursor protein cleaving enzyme)	Central causal role in Alzheimer's disease	[50]
nephrin promoter	Nephrin synthesis	[51]
CIDEA (Cell death-inducing DNA fragmentation factor alpha-like effector A)	Proapoptotic protein	[52]
TFF2 (Trefoil factor family 2)	Defense and repair of gastric mucosa	[53]

**Table 2:** PPAR-independent effects on tumor-related genes.

PPARs ligand	PPAR-independent effect	Experimental strategies	Ref.
*PPAR*γ* ligands*			
Troglitazone in LNCaP prostate cancer cells	Androgen receptor (AR) suppression by facilitating the ubiquitin-dependent proteasomal degradation of the transcriptional factor Sp-1	STG28, a PPAR*γ*-inactive analogue of troglitazone.	[143]
Troglitazone in mice	Rapidly AMP-activated protein kinase (AMPK) activation through a yet undefined PPAR-*γ*-independent mechanism, leading to the suppression of insulin-like growth factor-I tumor-promoting activity (IGF-1)	Expression of a dominant-negative AMPK	[144]
Troglitazone and ciglitazone in MCF-7 breast cancer	Repression of cyclin D1 expression, though a post transcriptional mechanism, via proteasome-facilitated proteolysis	Proteasome inhibitors	[145]
Ciglitazone in HT1080 human fibrosarcoma	Increase of MMP-2 expression through ROS production and ERK activation	PPAR*γ* antagonist GW9662	[146]
Troglitazone and 15-deoxy-prostaglandin J2 (15dPGJ2) in prostate and bladder cancer cells	Troglitazone induces G0/G1 growth arrest and PGJ2 induces apoptosis	PPAR*γ* antagonist GW9662	[147]
Troglitazone in B cell acute lymphoblastic leukemia cell lines	Apoptosis and cell growth inhibition associated with G1 cell cycle arrest	PPAR antagonists	[148]
Thiazolidinediones (TZD) in human breast cancer	Inhibition of Cyclin D3 expression by decreasing cyclin mRNA levels and by inducing its proteasomal degradation	A dominant negative mutant of PPAR*γ*	[149]
Troglitazone in mouse skin keratinocytes	Inhibition of cyclin D1 expression	PPAR*γ* antagonist GW9662 and dominant Dominant negative PPAR*γ*.	[150]
Thiazolidinediones (TGZ) in human colon cancer cells HTC-116	Egr-1 promoter activity increase	Different PPAR*γ* ligands	[140]
15-deoxy-prostaglandin J2 (15dPGJ2) in colon carcinoma cells	COX2 and VEGF inhibition via AP-1 activity repression	Dominant negative form of PPAR*γ* and a PPAR*γ* antagonist	[151]
15-deoxy-prostaglandin J2 (15dPGJ2) induces apoptosis in human B lymphocytes	Apoptosis through the induction of ROS and depletion of glutathione	Dominant negative form of PPAR*γ* and a PPAR*γ* antagonist	[152]
15-deoxy-prostaglandin J2 (15dPGJ2) in Jurkat human leukemic cells and PC3 human prostate cancer cells	Apoptosis by increasing the mRNA stability of death Receptor 5 (DR5), a specific receptor for tumor-necrosis factor-related apoptosis-inducing ligand (TRAIL)	PPAR*γ* antagonist GW9662	[153]

*PPAR*α* ligands*			
DEHP in mice	Induction of hepatic tumorigenesis	Wild-type and PPAR*α*-null mice in comparison	[154]
WY14,643 in activated splenocytes isolated from C57BL/6 mice	Apoptosis	Wild-type and PPAR*α*-null mice in comparison	[155]

*PPAR*β*/*δ* ligands*			
GW0742 in PPAR*β*-null mouse model	Induction of keratinocyte terminal differentiation and inhibition of keratinocyte proliferation	PPAR*δ*-null mice	[86]

## References

[1] Palmer CNA, Hsu M-H, Griffin KJ, Johnson EF (1995). Novel sequence determinants in peroxisome proliferator signaling. *The Journal of Biological Chemistry*.

[2] Hess R, Stäubli W, Riess W (1965). Nature of the hepatomegalic effect produced by ethyl-chlorophenoxy-isobutyrate in the rat. *Nature*.

[3] Willson TM, Brown PJ, Sternbach DD, Henke BR (2000). The PPARs: from orphan receptors to drug discovery. *Journal of Medicinal Chemistry*.

[4] Issemann I, Green S (1990). Activation of a member of the steroid hormone receptor superfamily by peroxisome proliferators. *Nature*.

[5] Cattley RC, DeLuca J, Elcombe C (1998). Do peroxisome proliferating compounds pose a hepatocarcinogenic hazard to humans?. *Regulatory Toxicology and Pharmacology*.

[6] Tontonoz P, Kim JB, Graves RA, Spiegelman BM (1993). ADD1: a novel helix-loop-helix transcription factor associated with adipocyte determination and differentiation. *Molecular and Cellular Biology*.

[7] Escher P, Braissant O, Basu-Modak S, Michalik L, Wahli W, Desvergne B (2001). Rat PPARs: quantitative analysis in adult rat tissues and regulation in fasting and refeeding. *Endocrinology*.

[8] Barish GD, Narkar VA, Evans RM (2006). PPAR*δ*: a dagger in the heart of the metabolic syndrome. *The Journal of Clinical Investigation*.

[9] Shulman AI, Mangelsdorf DJ (2005). Retinoid X receptor heterodimers in the metabolic syndrome. *The New England Journal of Medicine*.

[10] Kliewer SA, Xu HE, Lambert MH, Willson TM (2001). Peroxisome proliferator-activated receptors: from genes to physiology. *Recent Progress in Hormone Research*.

[11] Glass CK, Ogawa S (2006). Combinatorial roles of nuclear receptors in inflammation and immunity. *Nature Reviews Immunology*.

[12] Yang W, Rachez C, Freedman LP (2000). Discrete roles for peroxisome proliferator-activated receptor *γ* and retinoid X receptor in recruiting nuclear receptor coactivators. *Molecular and Cellular Biology*.

[13] Puigserver P, Spiegelman BM (2003). Peroxisome proliferator-activated receptor-*γ* coactivator 1*α* (PGC-1*α*): transcriptional coactivator and metabolic regulator. *Endocrine Reviews*.

[14] Kliewer SA, Umesono K, Noonan DJ, Heyman RA, Evans RM (1992). Convergence of 9-*cis* retinoic acid and peroxisome proliferator signalling pathways through heterodimer formation of their receptors. *Nature*.

[15] Palmer CNA, Hsu M-H, Muerhoff AS, Griffin KJ, Johnson EF (1994). Interaction of the peroxisome proliferator-activated receptor *α* with the retinoid X receptor *α* unmasks a cryptic peroxisome proliferator response element that overlaps an ARP-1-binding site in the *CYP4A6* promoter. *The Journal of Biological Chemistry*.

[16] Castelein H, Gulick T, Declercq PE, Mannaerts GP, Moore DD, Baes MI (1994). The peroxisome proliferator activated receptor regulates malic enzyme gene expression. *The Journal of Biological Chemistry*.

[17] Schoonjans K, Staels B, Auwerx J (1996). Role of the peroxisome proliferator-activated receptor (PPAR) in mediating the effects of fibrates and fatty acids on gene expression. *Journal of Lipid Research*.

[18] Schoonjans K, Peinado-Onsurbe J, Lefebvre A-M (1996). PPAR*α* and PPAR*γ* activators direct a distinct tissue-specific transcriptional response via a PPRE in the lipoprotein lipase gene. *The EMBO Journal*.

[19] Acín A, Rodriguez M, Rique H, Canet E, Boutin JA, Galizzi J-P (1999). Cloning and characterization of the 5′ flanking region of the human uncoupling protein 3 (UCP3) gene. *Biochemical and Biophysical Research Communications*.

[20] Ghosh S, Natarajan R (2001). Cloning of the human cholesteryl ester hydrolase promoter: identification of functional peroxisomal proliferator-activated receptor responsive elements. *Biochemical and Biophysical Research Communications*.

[21] Kassam A, Capone JP, Rachubinski RA (2001). The short heterodimer partner receptor differentially modulates peroxisome proliferator-activated receptor *α*-mediated transcription from the peroxisome proliferator-response elements of the genes encoding the peroxisomal *β*-oxidation enzymes acyl-CoA oxidase and hydratase-dehydrogenase. *Molecular and Cellular Endocrinology*.

[22] Di-Poï N, Tan NS, Michalik L, Wahli W, Desvergne B (2002). Antiapoptotic role of PPAR*β* in keratinocytes via transcriptional control of the Akt1 signaling pathway. *Molecular Cell*.

[23] Iida KT, Kawakami Y, Suzuki H (2002). PPAR*γ* ligands, troglitazone and pioglitazone, up-regulate expression of HMG-CoA synthase and HMG-CoA reductase gene in THP-1 macrophages. *FEBS Letters*.

[24] Gauthier A, Vassiliou G, Benoist F, McPherson R (2003). Adipocyte low density lipoprotein receptor-related protein gene expression and function is regulated by peroxisome proliferator-activated receptor *γ*. *The Journal of Biological Chemistry*.

[25] Baldán Á, Relat J, Marrero PF, Haro D (2004). Functional interaction between peroxisome proliferator-activated receptors-*α* and Mef-2C on human carnitine palmitoyltransferase 1*β* (CPT1*β*) gene activation. *Nucleic Acids Research*.

[26] Brouillette C, Bossé Y, Pérusse L, Gaudet D, Vohl M-C (2004). Effect of liver fatty acid binding protein (FABP) T94A missense mutation on plasma lipoprotein responsiveness to treatment with fenofibrate. *Journal of Human Genetics*.

[27] Targett-Adams P, McElwee MJ, Ehrenborg E, Gustafsson MC, Palmer CN, McLauchlan J (2005). A PPAR response element regulates transcription of the gene for human adipose differentiation-related protein. *Biochimica et Biophysica Acta*.

[28] Mandard S, Zandbergen F, Tan NS (2004). The direct peroxisome proliferator-activated receptor target fasting-induced adipose factor (FIAF/PGAR/ANGPTL4) is present in blood plasma as a truncated protein that is increased by fenofibrate treatment. *The Journal of Biological Chemistry*.

[29] Kim H, Cha J-Y, Kim S-Y (2002). Peroxisomal proliferator-activated receptor-*γ* upregulates glucokinase gene expression in *β*-cells. *Diabetes*.

[30] Patsouris D, Mandard S, Voshol PJ (2004). PPAR*α* governs glycerol metabolism. *The Journal of Clinical Investigation*.

[31] Vatsyayan J, Lin C-T, Peng H-L, Chang H-Y (2006). Identification of a *cis*-acting element responsible for negative regulation of the human UDP-glucose dehydrogenase gene expression. *Bioscience, Biotechnology and Biochemistry*.

[32] Degenhardt T, Saramäki A, Malinen M (2007). Three members of the human pyruvate dehydrogenase kinase gene family are direct targets of the peroxisome proliferator-activated receptor *β*/*δ*. *Journal of Molecular Biology*.

[33] Kim H, Koh Y-K, Kim T-H (2007). Transcriptional activation of SHP by PPAR-*γ* in liver. *Biochemical and Biophysical Research Communications*.

[34] Coyle AT, O'Keeffe MB, Kinsella BT (2005). 15-deoxy Δ^12,14^-prostaglandin J_2_ suppresses transcription by promoter 3 of the human thromboxane A_2_ receptor gene through peroxisome proliferator-activated receptor *γ* in human erythroleukemia cells. *FEBS Journal*.

[35] Stienstra R, Mandard S, Tan NS (2007). The Interleukin-1 receptor antagonist is a direct target gene of PPAR*α* in liver. *Journal of Hepatology*.

[36] Jedidi I, Couturier M, Thérond P (2006). Cholesteryl ester hydroperoxides increase macrophage CD36 gene expression via PPAR*α*. *Biochemical and Biophysical Research Communications*.

[37] Ravaux L, Denoyelle C, Monne C, Limon I, Raymondjean M, El Hadri K (2007). Inhibition of interleukin-1*β*-induced group IIA secretory phospholipase A2 expression by peroxisome proliferator-activated receptors (PPARs) in rat vascular smooth muscle cells: cooperation between PPAR*β* and the proto-oncogene *BCL-6*. *Molecular and Cellular Biology*.

[38] Villard PH, Caverni S, Baanannou A (2007). PPAR*α* transcriptionally induces AhR expression in Caco-2, but represses AhR pro-inflammatory effects. *Biochemical and Biophysical Research Communications*.

[39] Ignatenko NA, Babbar N, Mehta D, Casero RA, Gerner EW (2004). Suppression of polyamine catabolism by activated Ki-ras in human colon cancer cells. *Molecular Carcinogenesis*.

[40] Zandbergen F, Mandard S, Escher P (2005). The G_0_/G_1_ switch gene 2 is a novel PPAR target gene. *Biochemical Journal*.

[41] Peeters LLH, Vigne J-L, Tee MK, Zhao D, Waite LL, Taylor RN (2006). PPAR*γ* represses VEGF expression in human endometrial cells: implications for uterine angiogenesis. *Angiogenesis*.

[42] Degenhardt T, Matilainen M, Herzig K-H, Dunlop TW, Carlberg C (2006). The insulin-like growth factor-binding protein 1 gene is a primary target of peroxisome proliferator-activated receptors. *The Journal of Biological Chemistry*.

[43] Sérée E, Villard P-H, Pascussi J-M (2004). Evidence for a new human CYP1A1 regulation pathway involving PPAR-*α* and 2 PPRE sites. *Gastroenterology*.

[44] Park EY, Cho IJ, Kim SG (2004). Transactivation of the PPAR-responsive enhancer module in chemopreventive glutathione *S*-transferase gene by the peroxisome proliferator-activated receptor-*γ* and retinoid X receptor heterodimer. *Cancer Research*.

[45] Pandhare J, Cooper SK, Phang JM (2006). Proline oxidase, a proapoptotic gene, is induced by troglitazone: evidence for both peroxisome proliferator-activated receptor *γ*-dependent and -independent mechanisms. *The Journal of Biological Chemistry*.

[46] Billiet L, Furman C, Larigauderie G (2008). Enhanced VDUP-1 gene expression by PPAR*γ* agonist induces apoptosis in human macrophage. *Journal of Cellular Physiology*.

[47] Boulanger A, McLemore P, Copeland NG (2003). Identification of beta-carotene 15,15′-monooxygenase as a peroxisome proliferator-activated receptor target gene. *The FASEB Journal*.

[48] Landrier JF, Thomas C, Grober J (2005). The gene encoding the human ileal bile acid-binding protein (I-BABP) is regulated by peroxisome proliferator-activated receptors. *Biochimica et Biophysica Acta*.

[49] Efrati E, Arsentiev-Rozenfeld J, Zelikovic I (2005). The human paracellin-1 gene (*hPCLN-1*): renal epithelial cell-specific expression and regulation. *American Journal of Physiology*.

[50] Sastre M, Dewachter I, Rossner S (2006). Nonsteroidal anti-inflammatory drugs repress *β*-secretase gene promoter activity by the activation of PPAR*γ*. *Proceedings of the National Academy of Sciences of the United States of America*.

[51] Benigni A, Zoja C, Tomasoni S (2006). Transcriptional regulation of nephrin gene by peroxisome proliferator-activated receptor-*γ* agonist: molecular mechanism of the antiproteinuric effect of pioglitazone. *Journal of the American Society of Nephrology*.

[52] Viswakarma N, Yu S, Naik S (2007). Transcriptional regulation of Cidea, mitochondrial cell death-inducing DNA fragmentation factor *α*-like effector A, in mouse liver by peroxisome proliferator-activated receptor *α* and *γ*. *The Journal of Biological Chemistry*.

[53] Shimada T, Fujii Y, Koike T (2007). Peroxisome proliferator-activated receptor *γ* (PPAR*γ*) regulates trefoil factor family 2 (TFF2) expression in gastric epithelial cells. *The International Journal of Biochemistry & Cell Biology*.

[54] Krogsdam A-M, Nielsen CAF, Neve S (2002). Nuclear receptor corepressor-dependent repression of peroxisome-proliferator-activated receptor *δ*-mediated transactivation. *Biochemical Journal*.

[55] Shi Y, Hon M, Evans RM (2002). The peroxisome proliferator-activated receptor *δ*, an integrator of transcriptional repression and nuclear receptor signaling. *Proceedings of the National Academy of Sciences of the United States of America*.

[56] Wang LH, Yang XY, Zhang X (2004). Transcriptional inactivation of STAT3 by PPAR*γ* suppresses IL-6-responsive multiple myeloma cells. *Immunity*.

[57] Ghisletti S, Huang W, Ogawa S (2007). Parallel SUMOylation-dependent pathways mediate gene- and signal-specific transrepression by LXRs and PPAR*γ*. *Molecular Cell*.

[58] Lee C-H, Chawla A, Urbiztondo N, Liao D, Boisvert WA, Evans RM (2003). Transcriptional repression of atherogenic inflammation: modulation by PPAR*δ*. *Science*.

[59] Delerive P, Fruchart J-C, Staels B (2001). Peroxisome proliferator-activated receptors in inflammation control. *Journal of Endocrinology*.

[60] Willson TM, Wahli W (1997). Peroxisome proliferator-activated receptor agonists. *Current Opinion in Chemical Biology*.

[61] Ahmed W, Ziouzenkova O, Brown J (2007). PPARs and their metabolic modulation: new mechanisms for transcriptional regulation?. *Journal of Internal Medicine*.

[62] Krey G, Braissant O, L'Horset F (1997). Fatty acids, eicosanoids, and hypolipidemic agents identified as ligands of peroxisome proliferator-activated receptors by coactivator-dependent receptor ligand assay. *Molecular Endocrinology*.

[63] Kliewer SA, Sundseth SS, Jones SA (1997). Fatty acids and eicosanoids regulate gene expression through direct interactions with peroxisome proliferator-activated receptors *α* and *γ*. *Proceedings of the National Academy of Sciences of the United States of America*.

[64] Forman BM, Chen J, Evans RM (1997). Hypolipidemic drugs, polyunsaturated fatty acids, and eicosanoids are ligands for peroxisome proliferator-activated receptors *α* and *δ*. *Proceedings of the National Academy of Sciences of the United States of America*.

[65] Devchand PR, Keller H, Peters JM, Vazquez M, Gonzalez FJ, Wahli W (1996). The PPAR*α*-leukotriene B_4_ pathway to inflammation control. *Nature*.

[66] Sethi S, Ziouzenkova O, Ni H, Wagner DD, Plutzky J, Mayadas TN (2002). Oxidized omega-3 fatty acids in fish oil inhibit leukocyte-endothelial interactions through activation of PPAR*α*. *Blood*.

[67] Theocharis S, Margeli A, Vielh P, Kouraklis G (2004). Peroxisome proliferator-activated receptor-*γ* ligands as cell-cycle modulators. *Cancer Treatment Reviews*.

[68] Kliewer SA, Lenhard JM, Willson TM, Patel I, Morris DC, Lehmann JM (1995). A prostaglandin J_2_ metabolite binds peroxisome proliferator-activated receptor *γ* and promotes adipocyte differentiation. *Cell*.

[69] Komar CM (2005). Peroxisome proliferator-activated receptors (PPARs) and ovarian function—implications for regulating steroidogenesis, differentiation, and tissue remodeling. *Reproductive Biology and Endocrinology*.

[70] Brown JD, Plutzky J (2007). Peroxisome proliferator-activated receptors as transcriptional nodal points and therapeutic targets. *Circulation*.

[71] Forman BM, Chen J, Evans RM (1996). The peroxisome proliferator-activated receptors: ligands and activators. *Annals of the New York Academy of Sciences*.

[72] Staels B, Fruchart J-C (2005). Therapeutic roles of peroxisome proliferator-activated receptor agonists. *Diabetes*.

[73] Lehmann JM, Moore LB, Smith-Oliver TA, Wilkison WO, Willson TM, Kliewer SA (1995). An antidiabetic thiazolidinedione is a high affinity ligand for peroxisome proliferator-activated receptor *γ* (PPAR*γ*). *The Journal of Biological Chemistry*.

[74] Lehrke M, Lazar MA (2005). The many faces of PPAR*γ*. *Cell*.

[75] Lehmann JM, Lenhard JM, Oliver BB, Ringold GM, Kliewer SA (1997). Peroxisome proliferator-activated receptors *α* and *γ* are activated by indomethacin and other non-steroidal anti-inflammatory drugs. *The Journal of Biological Chemistry*.

[76] Adamson DJA, Frew D, Tatoud R, Wolf CR, Palmer CNA (2002). Diclofenac antagonizes peroxisome proliferator-activated receptor-*γ* signaling. *Molecular Pharmacology*.

[77] Leibowitz MD, Fiévet C, Hennuyer N (2000). Activation of PPAR*δ* alters lipid metabolism in db/db mice. *FEBS Letters*.

[78] Oliver WR, Shenk JL, Snaith MR (2001). A selective peroxisome proliferator-activated receptor *δ* agonist promotes reverse cholesterol transport. *Proceedings of the National Academy of Sciences of the United States of America*.

[79] He T-C, Chan TA, Vogelstein B, Kinzler KW (1999). PPAR*δ* is an APC-regulated target of nonsteroidal anti-inflammatory drugs. *Cell*.

[80] Xu L, Han C, Wu T (2006). A novel positive feedback loop between peroxisome proliferator-activated receptor-*δ* and prostaglandin E_2_ signaling pathways for human cholangiocarcinoma cell growth. *The Journal of Biological Chemistry*.

[81] Matthiessen MW, Pedersen G, Albrektsen T, Adamsen S, Fleckner J, Brynskov J (2005). Peroxisome proliferator-activated receptor expression and activation in normal human colonic epithelial cells and tubular adenomas. *Scandinavian Journal of Gastroenterology*.

[82] Fukumoto K, Yano Y, Virgona N (2005). Peroxisome proliferator-activated receptor *δ* as a molecular target to regulate lung cancer cell growth. *FEBS Letters*.

[83] Ali FY, Egan K, FitzGerald GA (2006). Role of prostacyclin versus peroxisome proliferator-activated receptor *β* receptors in prostacyclin sensing by lung fibroblasts. *American Journal of Respiratory Cell and Molecular Biology*.

[84] Planavila A, Rodríguez-Calvo R, Jové M (2005). Peroxisome proliferator-activated receptor *β*/*δ* activation inhibits hypertrophy in neonatal rat cardiomyocytes. *Cardiovascular Research*.

[85] Martinasso G, Maggiora M, Trombetta A, Canuto RA, Muzio G (2006). Effects of di(2-ethylhexyl) phthalate, a widely used peroxisome proliferator and plasticizer, on cell growth in the human keratino cyte cell line NCTC 2544. *Journal of Toxicology and Environmental Health, Part A*.

[86] Westergaard M, Henningsen J, Svendsen ML (2001). Modulation of keratinocyte gene expression and differentiation by PPAR-selective ligands and tetradecylthioacetic acid. *Journal of Investigative Dermatology*.

[87] Kim DJ, Bility MT, Billin AN, Willson TM, Gonzalez FJ, Peters JM (2006). PPAR*β*/*δ* selectively induces differentiation and inhibits cell proliferation. *Cell Death & Differentiation*.

[88] Nilsson A, Ostlund Farrants AK, Nesland JM, Finstad HS, Pedersen JI (1995). Potentiating effects of clofibric acid on the differentiation of HL-60 human promyelocytic leukemia cells induced by retinoids. *European Journal of Cell Biology*.

[89] Hanley K, Jiang Y, He SS (1998). Keratinocyte differentiation is stimulated by activators of the nuclear hormone receptor PPAR*α*. *Journal of Investigative Dermatology*.

[90] Bronfman M, Ponce C, Rojas S (1998). Enhanced differentiation of HL-60 leukemia cells to macrophages induced by ciprofibrate. *European Journal of Cell Biology*.

[91] Scatena R, Nocca G, Sole PD (1999). Bezafibrate as differentiating factor of human myeloid leukemia cells. *Cell Death & Differentiation*.

[92] Demetri GD, Fletcher CDM, Mueller E (1999). Induction of solid tumor differentiation by the peroxisome proliferator-activated receptor-*γ* ligand troglitazone in patients with liposarcoma. *Proceedings of the National Academy of Sciences of the United States of America*.

[93] Mueller E, Sarraf P, Tontonoz P (1998). Terminal differentiation of human breast cancer through PPAR*γ*. *Molecular Cell*.

[94] Pizzimenti S, Laurora S, Briatore F, Ferretti C, Dianzani MU, Barrera G (2002). Synergistic effect of 4-hydroxynonenal and PPAR ligands in controlling human leukemic cell growth and differentiation. *Free Radical Biology and Medicine*.

[95] Schmuth M, Haqq CM, Cairns WJ (2004). Peroxisome proliferator-activated receptor (PPAR)-*β*/*δ* stimulates differentiation and lipid accumulation in keratinocytes. *Journal of Investigative Dermatology*.

[96] Tan NS, Michalik L, Noy N (2001). Critical roles of PPAR*β*/*δ* in keratinocyte response to inflammation. *Genes & Development*.

[97] Aung CS, Faddy HM, Lister EJ, Monteith GR, Roberts-Thomson SJ (2006). Isoform specific changes in PPAR*α* and *β* in colon and breast cancer with differentiation. *Biochemical and Biophysical Research Communications*.

[98] Vosper H, Patel L, Graham TL (2001). The peroxisome proliferator-activated receptor *δ* promotes lipid accumulation in human macrophages. *The Journal of Biological Chemistry*.

[99] Saluja I, Granneman JG, Skoff RP (2001). PPAR *δ* agonists stimulate oligodendrocyte differentiation in tissue culture. *Glia*.

[100] Laurora S, Pizzimenti S, Briatore F (2003). Peroxisome proliferator-activated receptor ligands affect growth-related gene expression in 
human leukemic cells. *Journal of Pharmacology and Experimental Therapeutics*.

[101] Fajas L, Debril M-B, Auwerx J (2001). Peroxisome proliferator-activated receptor-*γ*: from adipogenesis to carcinogenesis. *Journal of Molecular Endocrinology*.

[102] Asou H, Verbeek W, Williamson E (1999). Growth inhibition of myeloid leukaemia cells by troglitazone, a ligand for peroxisome proliferator activated receptor gamma, and retinoids. *International Journal of Oncology*.

[103] Yin F, Wakino S, Liu Z (2001). Troglitazone inhibits growth of MCF-7 breast carcinoma cells by targeting G1 cell cycle regulators. *Biochemical and Biophysical Research Communications*.

[104] Morrison RF, Farmer SR (1999). Role of PPAR*γ* in regulating a cascade expression of cyclin-dependent kinase inhibitors, p18(INK4c) and p21(Waf1/Cip1), during adipogenesis. *The Journal of Biological Chemistry*.

[105] Giacinti C, Giordano A (2006). RB and cell cycle progression. *Oncogene*.

[106] Wakino S, Kintscher U, Kim S, Yin F, Hsueh WA, Law RE (2000). Peroxisome proliferator-activated receptor *γ* ligands inhibit retinoblastoma phosphorylation and G_1_→S transition in vascular smooth muscle cells. *The Journal of Biological Chemistry*.

[107] de Dios ST, Bruemmer D, Dilley RJ (2003). Inhibitory activity of clinical thiazolidinedione peroxisome proliferator activating receptor-*γ* ligands toward internal mammary artery, radial artery, and saphenous vein smooth muscle cell proliferation. *Circulation*.

[108] Bruemmer D, Berger JP, Liu J (2003). A non-thiazolidinedione partial peroxisome proliferator-activated receptor *γ* ligand inhibits vascular smooth muscle cell growth. *European Journal of Pharmacology*.

[109] Kawa S, Nikaido T, Unno H, Usuda N, Nakayama K, Kiyosawa K (2002). Growth inhibition and differentiation of pancreatic cancer cell lines by PPAR*γ* ligand troglitazone. *Pancreas*.

[110] Burdick AD, Bility MT, Girroir EE (2007). Ligand activation of peroxisome proliferator-activated receptor-*β*/*δ*(PPAR*β*/*δ*) inhibits cell growth of human N/Tyyy3-1 keratinocytes. *Cellular Signalling*.

[111] Chou F-S, Wang P-S, Kulp S, Pinzone JJ (2007). Effects of thiazolidinediones on differentiation, proliferation, and apoptosis. *Molecular Cancer Research*.

[112] Kim Y, Suh N, Sporn M, Reed JC (2002). An inducible pathway for degradation of FLIP protein sensitizes tumor cells to TRAIL-induced 
apoptosis. *The Journal of Biological Chemistry*.

[113] Shimada T, Kojima K, Yoshiura K, Hiraishi H, Terano A (2002). Characteristics of the peroxisome proliferator activated receptor *γ* (PPAR*γ*) ligand induced apoptosis in colon cancer cells. *Gut*.

[114] Toyoda M, Takagi H, Horiguchi N (2002). A ligand for peroxisome proliferator activated receptor *γ* inhibits cell growth and induces apoptosis in human liver cancer cells. *Gut*.

[115] Elstner E, Müller C, Koshizuka K (1998). Ligands for peroxisome proliferator-activated receptor*γ* and retinoic acid receptor inhibit growth and induce apoptosis of human breast cancer cells in vitro 
and in BNX mice. *Proceedings of the National Academy of Sciences of the United States of America*.

[116] Clay CE, Atsumi G, High KP, Chilton FH (2001). Early *de novo* gene expression is required for 15-deoxy-Δ^12,14^-prostaglandin J_2_-induced apoptosis in breast cancer cells. *The Journal of Biological Chemistry*.

[117] Kondo M, Shibata T, Kumagai T (2002). 15-deoxy-Δ^12,14^-prostaglandin J_2_: the endogenous electrophile that induces neuronal apoptosis. *Proceedings of the National Academy of Sciences of the United States of America*.

[118] Lin MS, Chen WC, Bai X, Wang YD (2007). Activation of peroxisome proliferator-activated receptor *γ* inhibits cell growth via apoptosis and arrest of the cell cycle in human colorectal cancer. *Journal of Digestive Diseases*.

[119] Liu H, Zang C, Fenner MH (2006). Growth inhibition and apoptosis in human Philadelphia chromosome-positive lymphoblastic leukemia cell lines by treatment with the dual PPAR*α*/*γ* ligand TZD18. *Blood*.

[120] Muzio G, Martinasso G, Trombetta A, Di Simone D, Canuto RA, Maggiora M (2006). HMG-CoA reductase and PPAR*α* are involved in clofibrate-induced apoptosis in human keratinocytes. *Apoptosis*.

[121] Shigeto T, Yokoyama Y, Xin B, Mizunuma H (2007). Peroxisome proliferator-activated receptor *α* and *γ* ligands inhibit the 
growth of human ovarian cancer. *Oncology Reports*.

[122] Muzio G, Maggiora M, Oraldi M, Trombetta A, Canuto RA (2007). PPAR*α* and PP2A are involved in the proapoptotic effect of conjugated linoleic acid on human hepatoma cell line 
SK-HEP-1. *International Journal of Cancer*.

[123] Gupta RA, Wang D, Katkuri S, Wang H, Dey SK, DuBois RN (2004). Activation of nuclear hormone receptor peroxisome proliferator-activated receptor-*δ* accelerates intestinal adenoma growth. *Nature Medicine*.

[124] Wang D, Wang H, Shi Q (2004). Prostaglandin E_2_ promotes colorectal adenoma growth via transactivation of the nuclear peroxisome proliferator-activated receptor *δ*. *Cancer Cell*.

[125] Michalik L, Desvergne B, Tan NS (2001). Impaired skin wound healing in peroxisome proliferator-activated receptor (PPAR)*α* and PPAR*β* mutant mice. *The Journal of Cell Biology*.

[126] Peters JM, Lee SST, Li W (2000). Growths, adipose, brain, and skin alterations resulting from targeted disruption of the mouse peroxisome proliferator-activated receptor *β*(*δ*). *Molecular and Cellular Biology*.

[127] Hatae T, Wada M, Yokoyama C, Shimonishi M, Tanabe T (2001). Prostacyclin-dependent apoptosis mediated by PPAR*δ*. *The Journal of Biological Chemistry*.

[128] Gupta RA, Brockman JA, Sarraf P, Willson TM, DuBois RN (2001). Target genes of peroxisome proliferator-activated receptor *γ* in colorectal cancer cells. *The Journal of Biological Chemistry*.

[129] Altiok S, Xu M, Spiegelman BM (1997). PPAR*γ* induces cell cycle withdrawal: inhibition of E2f/DP DNA-binding activity via down-regulation of PP2A. *Genes & Development*.

[130] Koeffler HP (2003). Peroxisome proliferator-activated receptor *γ* and cancers. *Clinical Cancer Research*.

[131] Yamakawa-Karakida N, Sugita K, Inukai T (2002). Ligand activation of peroxisome proliferator-activated receptor *γ* induces apoptosis of leukemia cells by down-regulating the c-*myc* gene expression via blockade of the 
Tcf-4 activity. *Cell Death & Differentiation*.

[132] Cerbone A, Toaldo C, Laurora S (2007). 4-hydroxynonenal and PPAR*γ* ligands affect proliferation, 
differentiation, and apoptosis in colon cancer cells. *Free Radical Biology and Medicine*.

[133] Bozzo F, Bocca C, Colombatto S, Miglietta A (2007). Antiproliferative effect of conjugated linoleic acid in caco-2 cells: involvement of PPAR*γ* and APC/*β*-catenin pathways. *Chemico-Biological Interactions*.

[134] Marui N, Sakai T, Hosokawa N (1990). N-*myc* suppression and cell cycle arrest at G_1_ phase by prostaglandins. *FEBS Letters*.

[135] Okura T, Nakamura M, Takata Y, Watanabe S, Kitami Y, Hiwada K (2000). Troglitazone induces apoptosis via the p53 and Gadd45 pathway in vascular smooth muscle cells. *European Journal of Pharmacology*.

[136] Palakurthi SS, Aktas H, Grubissich LM, Mortensen RM, Halperin JA (2001). Anticancer effects of thiazolidinediones are independent of peroxisome proliferator-activated receptor *γ* and mediated by inhibition of translation initiation. *Cancer Research*.

[137] Gouni-Berthold I, Berthold HK, Weber A-A (2001). Troglitazone and rosiglitazone induce apoptosis of vascular smooth muscle cells through an extracellular signal-regulated kinase-independent pathway. *Naunyn-Schmiedeberg's Archives of Pharmacology*.

[138] Takeda K, Ichiki T, Tokunou T, Iino N, Takeshita A (2001). 15-deoxy-Δ^12,14^-prostaglandin J_2_ and thiazolidinediones activate the MEK/ERK pathway through phosphatidylinositol 3-kinase in vascular smooth muscle cells. *The Journal of Biological Chemistry*.

[139] Bae M-A, Song BJ (2003). Critical role of c-Jun N-terminal protein kinase activation in troglitazone-induced apoptosis of human HepG2 hepatoma cells. *Molecular Pharmacology*.

[140] Baek SJ, Wilson LC, Hsi LC, Eling TE (2003). Troglitazone, a peroxisome proliferator-activated receptor *γ* (PPAR*γ*) ligand, selectively induces the early growth response-1 gene independently of PPAR*γ*. A novel mechanism for its anti-tumorigenic activity. *The Journal of Biological Chemistry*.

[141] Motomura W, Okumura T, Takahashi N, Obara T, Kohgo Y (2000). Activation of peroxisome proliferator-activated receptor *γ* by troglitazone inhibits cell growth through the increase of p27^Kip1^ in human pancreatic carcinoma cells. *Cancer Research*.

[142] Sugimura A, Kiriyama Y, Nochi H (1999). Troglitazone suppresses cell growth of myeloid leukemia cell lines by induction of p21WAF1/CIP1 cyclin-dependent kinase inhibitor. *Biochemical and Biophysical Research Communications*.

[143] Yang C-C, Wang Y-C, Wei S (2007). Peroxisome proliferator-activated receptor *γ*-independent suppression of androgen receptor expression by troglitazone mechanism and pharmacologic exploitation. *Cancer Research*.

[144] He G, Sung YM, DiGiovanni J, Fischer SM (2006). Thiazolidinediones inhibit insulin-like growth factor-I-induced activation of p70S6 kinase and suppress insulin-like growth factor-I tumor-promoting activity. *Cancer Research*.

[145] Huang J-W, Shiau C-W, Yang Y-T (2005). Peroxisome proliferator-activated receptor *γ*-independent ablation of cyclin D1 by thiazolidinediones and their derivatives in breast cancer cells. *Molecular Pharmacology*.

[146] Kim K-H, Cho YS, Park J-M, Yoon S-O, Kim K-W, Chung A-S (2007). Pro-MMP-2 activation by the PPAR*γ* agonist, ciglitazone, induces cell invasion through the generation of ROS and the activation of ERK. *FEBS Letters*.

[147] Chaffer CL, Thomas DM, Thompson EW, Williams ED (2006). PPAR*γ*-independent induction of growth arrest and apoptosis in prostate and bladder carcinoma. *BMC Cancer*.

[148] Takenokuchi M, Saigo K, Nakamachi Y (2006). Troglitazone inhibits cell growth and induces apoptosis of B-cell acute lymphoblastic leukemia cells with 
t(14;18). *Acta Haematologica*.

[149] Lu M, Kwan T, Yu C (2005). Peroxisome proliferator-activated receptor *γ* agonists promote TRAIL-induced apoptosis by reducing survivin levels via cyclin D3 repression and cell cycle arrest. *The Journal of Biological Chemistry*.

[150] He G, Thuillier P, Fischer SM (2004). Troglitazone inhibits cyclin D1 expression and cell cycling independently of PPAR*γ* in normal mouse skin keratinocytes. *Journal of Investigative Dermatology*.

[151] Grau R, Iñiguez MA, Fresno M (2004). Inhibition of activator protein 1 activation, vascular endothelial growth factor, and cyclooxygenase-2 expression by 15-deoxy-Δ^12,14^- prostaglandin J_2_ in colon carcinoma cells: evidence for a redox-sensitive peroxisome proliferator-activated receptor-*γ*-independent mechanism. *Cancer Research*.

[152] Ray DM, Akbiyik F, Phipps RP (2006). The peroxisome proliferator-activated receptor *γ* (PPAR*γ*) ligands 15-deoxy-Δ^12,14^-prostaglandin J_2_ and ciglitazone induce human B lymphocyte and B cell lymphoma apoptosis by PPAR*γ*-independent mechanisms. *The Journal of Immunology*.

[153] Nakata S, Yoshida T, Shiraishi T (2006). 15-deoxy-Δ^12,14^-prostaglandin J_2_ induces death receptor 5 expression through mRNA stabilization independently of PPAR*γ* and potentiates TRAIL-induced apoptosis. *Molecular Cancer Therapeutics*.

[154] Ito Y, Yamanoshita O, Asaeda N (2007). Di(2-ethylhexyl)phthalate induces hepatic tumorigenesis through a peroxisome proliferator-activated receptor 
*α*-independent pathway. *Journal of Occupational Health*.

[155] Cunard R, DiCampli D, Archer DC (2002). WY14,643, a PPAR*α* ligand, has profound effects on immune responses in vivo. *The Journal of Immunology*.

[156] Srivastava S, Conklin DJ, Liu S-Q (2001). Identification of biochemical pathways for the metabolism of oxidized low-density lipoprotein derived aldehyde-4-hydroxy *trans*-2-nonenal in vascular smooth muscle cells. *Atherosclerosis*.

[157] Dianzani MU (2003). 4-hydroxynonenal from pathology to physiology. *Molecular Aspects of Medicine*.

[158] Esterbauer H, Schaur RJ, Zollner H (1991). Chemistry and biochemistry of 4-hydroxynonenal, malonaldehyde and related aldehydes. *Free Radical Biology and Medicine*.

[159] Uchida K (2003). 4-hydroxy-2-nonenal: a product and mediator of oxidative stress. *Progress in Lipid Research*.

[160] Awasthi YC, Ansari GAS, Awasthi S (2005). Regulation of 4-hydroxynonenal mediated signaling by glutathione S-transferases. *Methods in Enzymology*.

[161] Yang Y, Sharma S, Sharma A, Awasthi S, Awasthi YC (2003). Lipid peroxidation and cell cycle signaling: 4-hydroxynonenal, a key molecule in stress mediated 
signalling. *Acta Biochimica Polonica*.

[162] Barrera G, Di Mauro C, Muraca R (1991). Induction of differentiation in human HL-60 cells by 4-hydroxynonenal, a 
product of lipid peroxidation. *Experimental Cell Research*.

[163] Barrera G, Pizzimenti S, Muraca R (1996). Effect of 4-hydroxynonenal on cell cycle progression and expression of differentiation-associated antigens in HL-60 cells. *Free Radical Biology and Medicine*.

[164] Awasthi YC, Sharma R, Cheng JZ (2003). Role of 4-hydroxynonenal in stress-mediated apoptosis signalling. *Molecular Aspects of Medicine*.

[165] Laurora S, Tamagno E, Briatore F (2005). 4-hydroxynonenal modulation of p53 family gene expression in the SK-N-BE neuroblastoma cell line. *Free Radical Biology and Medicine*.

[166] Pizzimenti S, Barrera G, Dianzani MU, Brüsselbach S (1999). Inhibition of D1, D2, and A-cyclin expression in HL-60 cells by the lipid peroxydation 
product 4-hydroxynonenal. *Free Radical Biology and Medicine*.

[167] Barrera G, Pizzimenti S, Laurora S, Moroni E, Giglioni B, Dianzani MU (2002). 4-hydroxynonenal affects pRb/E2F pathway in HL-60 human leukemic cells. *Biochemical and Biophysical Research Communications*.

[168] Pizzimenti S, Briatore F, Laurora S (2006). 4-hydroxynonenal inhibits telomerase activity and hTyyy3 expression in human leukemic cell lines. *Free Radical Biology and Medicine*.

[169] Tontonoz P, Nagy L, Alvarez JGA, Thomaszy VA, Evans RM (1998). PPAR-*γ* promotes monocyte/magrophage differentiation and uptake of oxidized LDL. *Cell*.

[170] Kliewer SA, Lenhard JM, Willson TM, Patel I, Morris DC, Lehmann JM (1995). A prostaglandin J_2_ metabolite binds peroxisome proliferator-activated receptor *γ* and promotes adipocyte differentiation. *Cell*.

[171] Iwashima Y, Eto M, Horiuchi S, Sano H (1999). Advanced glycation end product-induced peroxisome proliferator-activated receptor *γ* gene expression in the cultured mesangial cells. *Biochemical and Biophysical Research Communications*.

[172] Kondo M, Oya-Ito T, Kumagai T, Osawa T, Uchida K (2001). Cyclopentenone prostaglandins as potential inducers of intracellular oxidative stress. *The Journal of Biological Chemistry*.

[173] Yeldandi AV, Rao MS, Reddy JK (2000). Hydrogen peroxide generation in peroxisome proliferator-induced oncogenesis. *Mutation Research*.

[174] Muzio G, Trombetta A, Maggiora M (2006). Arachidonic acid suppresses growth of human lung tumor A549 cells through down-regulation of ALDH3A1 expression. *Free Radical Biology and Medicine*.

[175] Coleman JD, Prabhu KS, Thompson JT (2007). The oxidative stress mediator 4-hydroxynonenal is an intracellular agonist of the nuclear receptor peroxisome proliferator-activated receptor-*β*/*δ* (PPAR*β*/*δ*). *Free Radical Biology and Medicine*.

